# Diagnostic performance of quantitative perfusion cardiac magnetic resonance imaging in patients with prior coronary artery disease

**DOI:** 10.1093/ehjci/jeae262

**Published:** 2024-10-09

**Authors:** Roel Hoek, Sonia Borodzicz-Jazdzyk, Pepijn A van Diemen, Yvemarie B O Somsen, Ruben W de Winter, Ruurt A Jukema, Jos W R Twisk, Pieter G Raijmakers, Juhani Knuuti, Teemu Maaniitty, S Richard Underwood, Eike Nagel, Lourens F H J Robbers, Ahmet Demirkiran, Martin B von Bartheld, Roel S Driessen, Ibrahim Danad, Marco J W Götte, Paul Knaapen

**Affiliations:** Department of Cardiology, Amsterdam University Medical Center, Vrije Universiteit Amsterdam, De Boelelaan 1117, Amsterdam 1081 HV, The Netherlands; Department of Cardiology, Amsterdam University Medical Center, Vrije Universiteit Amsterdam, De Boelelaan 1117, Amsterdam 1081 HV, The Netherlands; First Department of Cardiology, Medical University of Warsaw, Warsaw, Poland; Department of Cardiology, Amsterdam University Medical Center, Vrije Universiteit Amsterdam, De Boelelaan 1117, Amsterdam 1081 HV, The Netherlands; Department of Cardiology, Amsterdam University Medical Center, Vrije Universiteit Amsterdam, De Boelelaan 1117, Amsterdam 1081 HV, The Netherlands; Department of Cardiology, Amsterdam University Medical Center, Vrije Universiteit Amsterdam, De Boelelaan 1117, Amsterdam 1081 HV, The Netherlands; Department of Cardiology, Amsterdam University Medical Center, Vrije Universiteit Amsterdam, De Boelelaan 1117, Amsterdam 1081 HV, The Netherlands; Department of Epidemiology & Data Science, Amsterdam University Medical Center, Vrije Universiteit Amsterdam, Amsterdam, The Netherlands; Department of Radiology & Nuclear Medicine, Amsterdam University Medical Center, Vrije Universiteit Amsterdam, Amsterdam, The Netherlands; Department of Clinical Physiology, Nuclear Medicine and PET and Turku PET Centre, Turku University Hospital, Turku, Finland; Department of Clinical Physiology, Nuclear Medicine and PET and Turku PET Centre, Turku University Hospital, Turku, Finland; Department of Nuclear Medicine, Royal Brompton Hospital, London, UK; Institute for Experimental and Translational Cardiovascular Imaging, University Hospital Frankfurt, Frankfurt am Main, Germany; Department of Cardiology, Amsterdam University Medical Center, Vrije Universiteit Amsterdam, De Boelelaan 1117, Amsterdam 1081 HV, The Netherlands; Department of Cardiology, Amsterdam University Medical Center, Vrije Universiteit Amsterdam, De Boelelaan 1117, Amsterdam 1081 HV, The Netherlands; Department of Cardiology, St Jansdal Hospital, Harderwijk, The Netherlands; Department of Cardiology, Amsterdam University Medical Center, Vrije Universiteit Amsterdam, De Boelelaan 1117, Amsterdam 1081 HV, The Netherlands; Department of Cardiology, Radboud University Medical Center, Nijmegen, The Netherlands; Department of Cardiology, Northwest Clinics, Alkmaar, The Netherlands; Department of Cardiology, Amsterdam University Medical Center, Vrije Universiteit Amsterdam, De Boelelaan 1117, Amsterdam 1081 HV, The Netherlands; Department of Cardiology, Amsterdam University Medical Center, Vrije Universiteit Amsterdam, De Boelelaan 1117, Amsterdam 1081 HV, The Netherlands

**Keywords:** quantitative perfusion cardiac magnetic resonance imaging, myocardial perfusion imaging, positron emission tomography, fractional flow reserve

## Abstract

**Aims:**

The diagnostic performance of quantitative perfusion cardiac magnetic resonance (QP-CMR) imaging has scarcely been evaluated in patients with a history of coronary artery disease (CAD) and new onset chest pain. The present study compared the diagnostic performance of automated QP-CMR for the detection of fractional flow reserve (FFR) defined hemodynamically significant CAD with visual assessment of first-pass stress perfusion CMR (v-CMR) and quantitative [^15^O]H_2_O positron emission tomography (PET) imaging in a true head-to-head fashion in patients with prior CAD.

**Methods and results:**

This PACIFIC-2 substudy included 145 symptomatic chronic coronary symptom patients with prior myocardial infarction and/or percutaneous coronary intervention. All patients underwent dual-sequence, single-bolus perfusion CMR, and [^15^O]H_2_O PET perfusion imaging followed by invasive coronary angiography with three-vessel FFR. Hemodynamically significant CAD was defined as an FFR ≤ 0.80. QP-CMR, v-CMR, and PET exhibited a sensitivity of 66, 67, and 80%, respectively, whereas specificity was 60, 62, and 63%. Sensitivity of QP-CMR was lower than that of PET (*P* = 0.015), whereas the specificity of QP-CMR and PET was comparable. Diagnostic accuracy and area under the curve of QP-CMR (64% and 0.66) was comparable to both v-CMR [66% (*P* = not significant [NS]) and 0.67 (*P* = NS)] and PET [74% (*P* = NS) and 0.78 (*P* = NS)].

**Conclusion:**

In patients with prior myocardial infarction and/or percutaneous coronary intervention, the diagnostic performance of QP-CMR was comparable to visual assessment of first-pass stress perfusion CMR and quantitative [^15^O]H_2_O PET for the detection of hemodynamically significant CAD as defined by FFR.

## Graphical Abstract

**Graphical Abstract:**
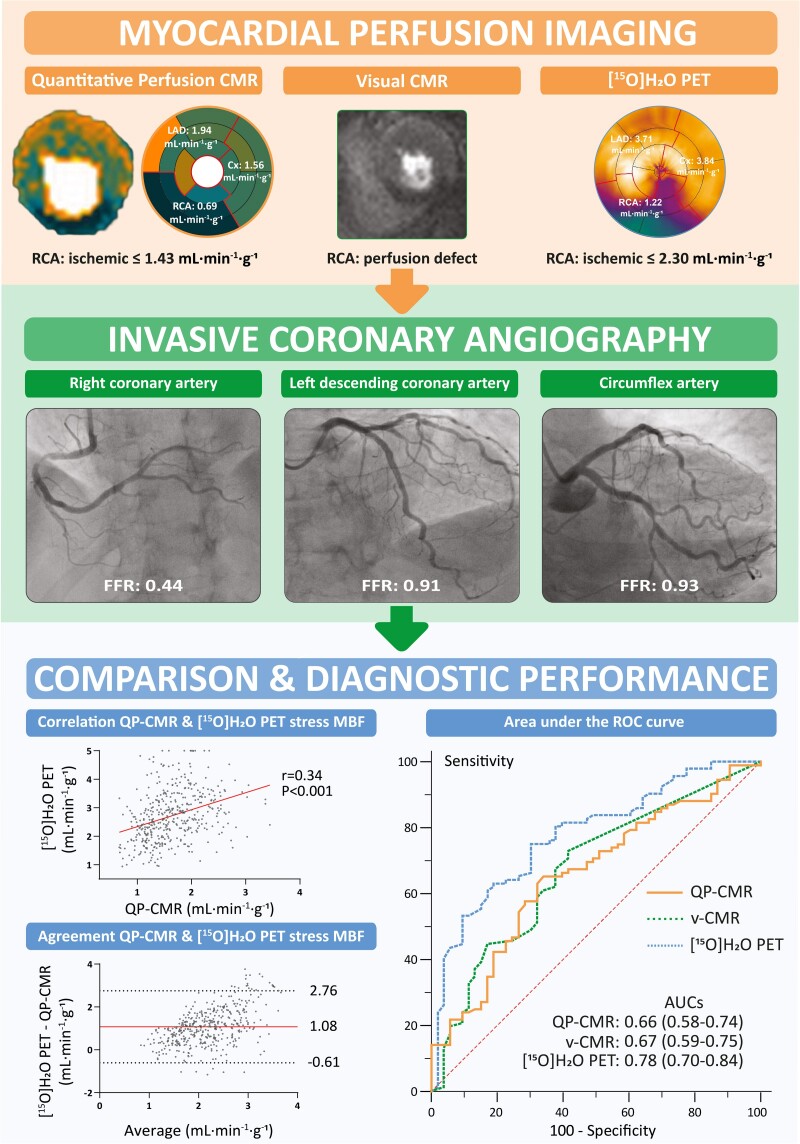

## Introduction

In patients with a history of coronary artery disease (CAD) and new onset chest pain, guidelines recommend myocardial perfusion imaging (MPI) to diagnose myocardial ischaemia and guide appropriate referral for invasive coronary angiography (ICA). However, recommendations on selecting a specific MPI modality are limited to local expertise and availability.^1,2^ Imaging modalities that allow for the quantification of myocardial blood flow (MBF) have demonstrated superior diagnostic performance over qualitative approaches.^3,4^ An emerging and promising technique in the field of quantitative MPI is quantitative perfusion cardiac magnetic resonance (QP-CMR) imaging, which has shown similar diagnostic performance as compared with ^13^NH_3_ positron emission tomography (PET) when referenced by quantitative coronary angiography.^5–8^ Moreover, QP-CMR outperforms visual assessment of first-pass stress perfusion CMR (v-CMR) in the detection of multivessel disease (MVD), suggesting a potential additive value of QP-CMR to CMR perfusion imaging in patients with known history of CAD.^9,10^ To date, a head-to-head comparison of QP-CMR vs. v-CMR and [^15^O]H_2_O PET in this challenging population is lacking. Therefore, in this PACIFIC-2^11^ substudy, we aimed to explore the diagnostic performance of automated QP-CMR for the detection of hemodynamically significant CAD defined by fractional flow reserve (FFR) as compared with v-CMR and [^15^O]H_2_O PET perfusion imaging in patients with prior myocardial infarction (MI) and/or percutaneous coronary intervention (PCI).

## Methods

### Patient population

This is a substudy of the PACIFIC-2 (Functional stress imaging to predict abnormal coronary fractional flow reserve) trial.^11^ PACIFIC-2 is a prospective clinical single-centre comparative study on the diagnostic performance of CMR, cardiac PET, and single-photon emission computed tomography perfusion imaging in 189 patients with a history of MI and/or PCI, with new symptoms suggestive of obstructive CAD. After MPI, all patients underwent ICA in conjunction with FFR interrogation of all major coronary arteries. In- and exclusion criteria of PACIFIC-2 have been previously described.^11^ In the present substudy, we included all patients in which stress perfusion CMR imaging was amenable to QP-CMR analysis. The PACIFIC-2 study was approved by the Institutional Medical Ethics Committee and complied with the Declaration of Helsinki. All patients provided written informed consent.

### Image acquisition

Non-invasive cardiac imaging was planned within 2 weeks prior to ICA. Patients were instructed to withhold from caffeine or xanthine-containing product 24 h prior to the scans and to be in a fasting state. Medication was continued without changes during the study protocol.

### Cardiac magnetic resonance imaging

CMR images were acquired using a 1.5 T whole-body MR scanner (Magnetom Avanto, Siemens Healthineers). Perfusion imaging was performed using a dual-sequence, single-bolus technique.^12^ First-pass stress perfusion imaging was performed during continuous intravenous infusion of adenosine (fixed at 140 μg/kg/min), followed by rest perfusion imaging 15 min after stress imaging. Myocardial perfusion images were acquired at basal, mid-ventricular, and apical levels in short-axis slices after a bolus of gadolinium-based contrast agent (GBCA) at a dose of 0.075 mmol/kg (DOTAREM, Guerbet, Villepinte, France). Left ventricular (LV) systolic function was assessed using steady-state free-precession cine imaging. Late gadolinium enhancement (LGE) images were acquired 12–15 min after rest perfusion using a 2D segmented inversion-recovery gradient echo pulse sequence. Quantitative analysis of CMR perfusion imaging was performed using Circle Cardiovascular Imaging 42 software (cvi42; Circle Cardiovascular Imaging Inc., Calgary, Canada), allowing automated pixel-wise QP mapping. This method has demonstrated good agreement with the manual delineation of regions of interest (ROI).^13^ First, stress and rest images without motion correction (MOCO) were selected. Next, the system automatically performed MOCO, and detected the arterial input function (AIF) and signal intensity (SI) curves of the GBCA. Manual corrections for the start of the contrast input and key time points on these curves were not possible, in case of suspicion of an error. Endo- and epicardial borders of the LV myocardium were delineated, correcting for papillary muscles and epicardial fat. If required, manual ROI adjustments were allowed. Subsequently, quantitative MBF (mL·min^−1^·g^−1^) maps were generated for each pixel and according to the standardized American Heart Association (AHA) 17-segment model, excluding the apex, through a reverse process of deconvolution of the myocardium’s SI curves and the AIF curve.^13–15^ Cases with the absence of the splenic switch-off phenomenon together with an increase in heart rate of < 10 bpm between rest and stress were considered a suboptimal hyperaemic response, and thus excluded in further analysis. All segments of the AHA 17-segment model, excluding the apex, were assigned to their respective vascular territory. In the case of a left dominant system, segments of the right coronary artery (RCA) were assigned to the circumflex artery (Cx), and the RCA was excluded for further analysis. The optimal cut-off calculation for detecting hemodynamically significant CAD, as indicated by an FFR ≤ 0.80, was performed on the average stress MBF of an entire coronary vascular territory. Ischaemia was assumed if significant hypoperfusion was present, defined as an average stress MBF of the entire vascular territory equal to or below the calculated cut-off. Twenty cases were analysed by two observers (R.H. and S.B.J.), blinded to the results of the other assessor. V-CMR analysis was performed under the supervision of a core laboratory (University Hospital Frankfurt, Frankfurt am Main, Germany). Stress and LGE images were visually analysed according to the AHA 17-segment model excluding the apex.^14^ The extent of stress perfusion defect and LGE were scored for each segment using a 5-point scale (0, 1–25, 26–50, 51–75, and > 75%). Ischaemia was assumed if significant hypoperfusion was present, defined as a perfusion defect extending beyond an area with LGE. In the absence of LGE, ischaemia was defined as a perfusion defect ≥ 2 segment circumferential, a perfusion defect extending ≥ 2 slice, or as a perfusion defect with > 50% transmurality. Vascular perfusion defect scores were calculated by subtracting the total LGE score from the total perfusion defect score (with a minimum score of 0) within a coronary territory. A vascular LGE score ≥ 2 was defined as significant scar and used for stratifying vessels in the corresponding subanalysis. An additional description of the image acquisition protocol and v-CMR analysis has been previously published.^11^

### [^15^O]H_2_O PET

PET perfusion images were acquired on a PET/CT device (Philips Gemini TF 64 or Ingenuity TF 128, Philips Healthcare, Best, The Netherlands) using 370 MBq of [^15^O]H_2_O during rest and stress, induced by continuous intravenous infusion of adenosine (fixed at 140 μg/kg/min). Reconstructed PET data was transferred to a core laboratory (Turku University Hospital, Turku, Finland), where images were analysed and interpreted by observers blinded to CMR and ICA/FFR results. Parametric MBF (mL·min^−1^·g^−1^) images were constructed and quantitatively analysed. MBF was calculated for all coronary segments according to the AHA 17-segment model at baseline and during hyperaemia.^14^ Again, all segments were assigned to their respective vascular territory, incorporating left dominant systems. Ischaemia was assumed if significant hypoperfusion was present, and was defined as stress MBF ≤ 2.3 mL·min^−1^·g^−1^ in ≥ 2 adjacent segments within a vascular territory.^3,16^ Extensive description of the image acquisition protocol and analysis has been previously published.^11^

### ICA and FFR

ICA was performed following a standard protocol in which at least two orthogonal projections were acquired for each major coronary artery. Intracoronary nitroglycerine (0.2 mL) was administered prior to contrast injection to induce maximal vasodilatation. After inducing maximal hyperaemia by infusion of intracoronary (150 μg) or intravenous (140 μg/kg/min) adenosine, FFR was calculated by dividing the mean distal intracoronary pressure by the mean arterial pressure. FFR was obtained in all major coronary arteries except for arteries in which FFR measurement was not possible due to stenosis severity. Hemodynamically significant CAD was defined as an FFR ≤ 0.80, or coronary lesions with diameter stenosis ≥ 90% if FFR was missing.

### Follow-up

Patient follow-up was conducted between 2022 and 2023 utilizing electronic medical records and telephonic follow-up. The primary outcome during follow-up was a combined endpoint comprising all-cause mortality and non-fatal MI. The occurrence of the first event was considered as the patient’s event. Follow-up duration was terminated at 72 months.

### Statistical analysis

Continuous variables are presented as mean ± standard deviation (SD) for normally distributed data, otherwise as median with inter-quartile range. The distribution of continuous variables was verified using histogram graphs and Q–Q plots. Categorical variables are presented as frequencies with percentages. Means of MBF and heart rate derived from CMR and PET for all patients were compared with the paired sample’s *t*-test. Pearson correlation and Bland–Altman analysis were used to compare quantitative modalities, excluding the apex of PET to equalize target territory. Correlations between FFR and the corresponding mean stress MBF of the coronary segments used for ischaemia assessment by QP-CMR and PET were performed using Pearson correlation as well, assigning FFR of 0.50 to occluded vessels in which FFR was missing. The optimal cut-off value for stress MBF of QP-CMR was calculated using the Youden Index. Per-vessel inter-observer variability was assessed using Bland–Altman analysis and intraclass correlation coefficients with a two-way mixed model for absolute measures. Areas under the receiver operating characteristics curves (AUCs) were constructed using stress MBF for QP-CMR and PET, and vascular perfusion defect score for v-CMR. AUCs were compared using the DeLong method. Per-patient diagnostic performance measures [sensitivity, specificity, negative predictive value (NPV), positive predictive value (PPV) and accuracy] were calculated including a 95% confidence interval (CI). A comparison of sensitivity, specificity and accuracy between the three modalities was performed using McNemar’s test. Sensitivity and specificity were compared in patients with and without obstructive CAD, respectively, whereas accuracy was compared in all patients. For the comparison of NPV and PPV, selecting patients based on above-mentioned filters is not sufficient, as both comparisons require the inclusion of patients with and without obstructive CAD. Therefore, a marginal regression model including an independent working correlation structure was used. Per-vessel diagnostic performance measurements were compared using generalized estimation equations with an exchangeable (sensitivity, specificity, and accuracy) or independent (NPV and PPV) correlation structure, to correct for multiple vessels deriving from the same patient. Per-vessel subanalysis was performed by stratifying patients for the presence of MVD (two- or three-vessel disease) and the presence of scar in a vascular region. Survival analysis was performed to compare ischaemic and non-ischaemic patients, as determined by the various diagnostic modalities. Kaplan–Meier curves were generated to evaluate event-free survival. The prognostic significance of ischaemia was assessed using hazard ratios (HR) and log-rank tests. A two-sided *P*-value < 0.05 was considered statistically significant, although for diagnostic performance measurements, a Bonferroni correction was applied for three pairwise comparisons between modalities. Statistical analyses were performed in the SPSS software package (IBM SPSS statistics 26.0, Armonk, NY, USA), Rstudio software package (Rstudio 4.0.3, 1.3.1093, Boston, MA, USA), and MedCalc Statistical Software version 20.006 (MedCalc Software bv, Ostend, Belgium).

## Results

### Study population

**Figure 1:**
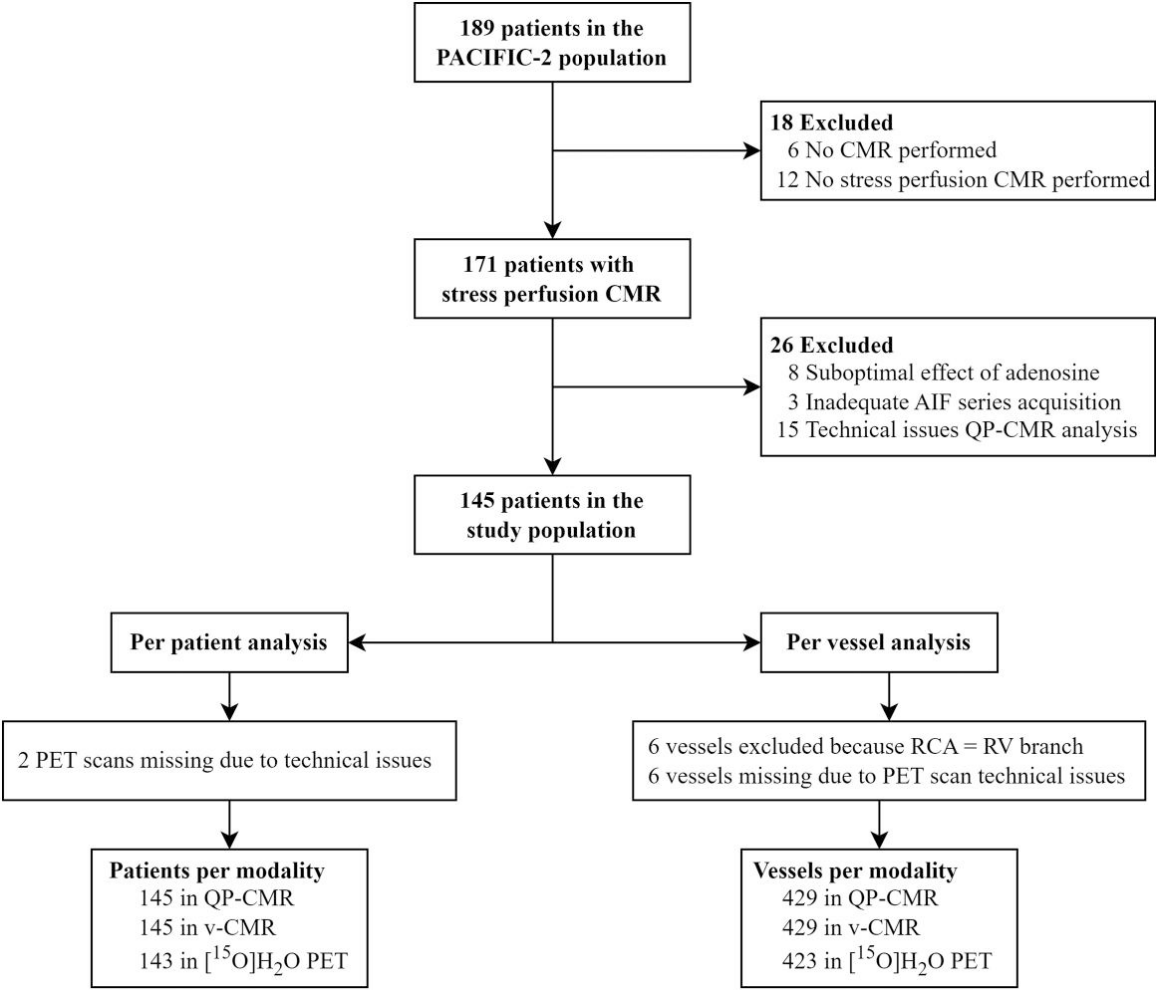
Study flowchart. Study flowchart demonstrating the included patient population and reason for exclusion in the present PACIFIC-2 substudy. AIF, arterial input function; CMR, cardiac magnetic resonance imaging; QP-CMR, quantitative perfusion CMR; v-CMR, visually assessed first-pass stress perfusion CMR; PET, positron emission tomography; RCA, right coronary artery; RV, right ventricular.

Of the 189 patients in the PACIFIC-2 population, 171 (90%) underwent stress perfusion CMR and were evaluated for inclusion in this substudy. Of these patients, 8 (5%) showed the suboptimal effect of adenosine, whereas QP-CMR analysis could not be performed due to inadequate AIF series acquisition during CMR scanning in 3 (2%) patients and due to technical issues of the software in 15 (9%) patients. As a result, this substudy included a total of 145 patients. For 6 patients, the RCA was functioning as a right ventricular branch and was thus excluded from the per-vessel analysis, resulting in a total of 429 vessels. For PET analysis, two additional patients (1%) were excluded due to PET acquisition failure. An overview of excluded patients and vessels is shown in the study flowchart (*Figure 1*). Baseline characteristics are listed in Table 1. In this study, 120 (83%) were male with a mean age of 64.2 ± 9.2 years. A total of 75 (52%) patients experienced a previous MI, and prior PCI was reported in 130 (90%) patients. Finally, based on ICA and subsequent FFR, 53 (37%) had no significant CAD, 54 (37%) had single-vessel disease, 31 (21%) had two-vessel disease and 7 (5%) had 3-vessel disease. FFR was successfully obtained in 393 out of 429 vessels (92%).

**Table 1:** Patient characteristics

	*n* = 145
Patient characteristics	
Age (years)	64.2 ± 9.2
Male gender	120 (83)
Body mass index (kg·m^−2^)	27.3 ± 3.9
Cardiac history	
Prior MI^a^	75 (52)
Prior PCI^a^	130 (90)
LVEF (%)^b^	58 ± 9
≥ 55	104 (72)
45–54	27 (19)
35–44	10 (7)
< 35	4 (3)
Cardiovascular risk factors	
Family history of CAD	77 (53)
Current smoker	17 (12)
History of smoking	60 (41)
Diabetes mellitus	32 (22)
Hypertension	95 (66)
Hypercholesterolemia	105 (72)
Symptoms	
Typical angina	58 (40)
Atypical angina	60 (41)
Non-specific chest discomfort	27 (19)
Medication	
Single antiplatelet therapy	91 (63)
Dual antiplatelet therapy	54 (37)
β-blocker	83 (57)
ACE-inhibitor or AR-blocker	83 (57)
Calcium channel blocker	47 (32)
Long acting nitrate	41 (28)
Statin	123 (85)
Angiographic characteristics	
No significant CAD	53 (37)
1 VD	54 (37)
2 VD	31 (21)
3 VD	7 (5)

Values are presented as mean ± SD or absolute numbers with corresponding percentages in parentheses.

^a^Prior MI and PCI as defined by documented cardiac history.

^b^LVEF as measured on CMR.

ACE, angiotensin-converting enzyme; AR, angiotensin receptor; CAD, coronary artery disease; CMR, cardiac magnetic resonance imaging; LVEF, left ventricular ejection fraction; MI, myocardial infarction; VD, vessel disease; PCI, percutaneous coronary intervention.

### Parameters of QP-CMR and [^15^O]H_2_O PET

**Figure 2:**
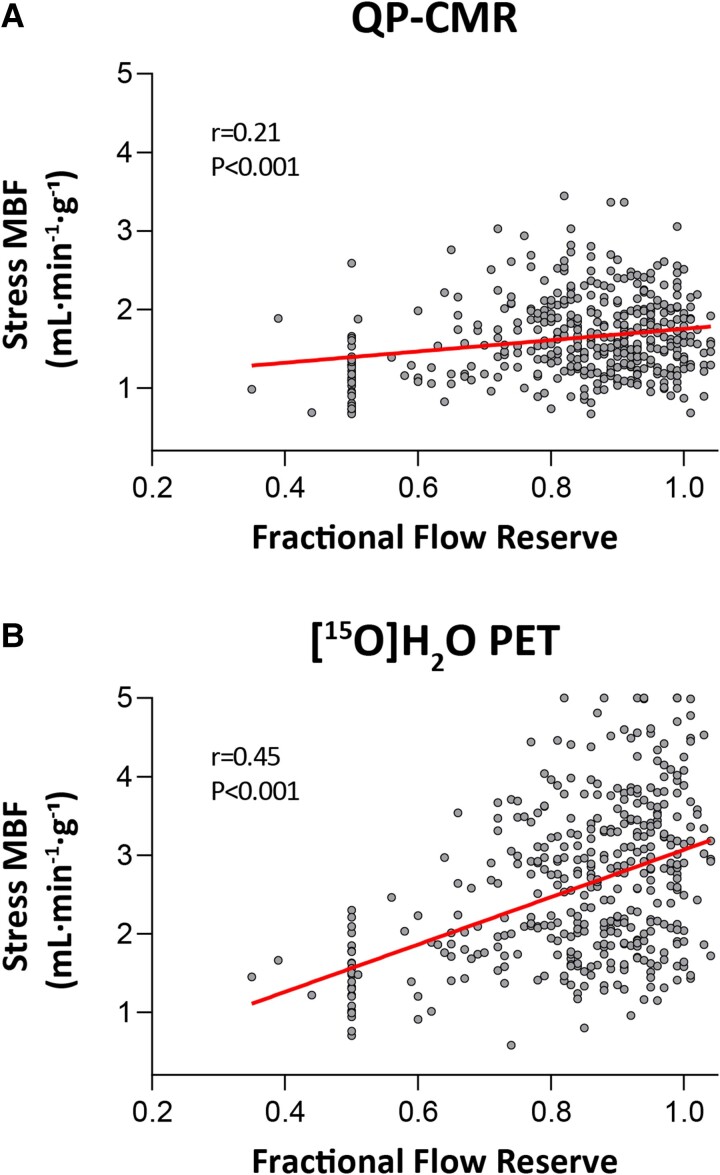
Correlation between FFR and quantitative stress MBF. Correlation between FFR and stress MBF of (*A*) QP-CMR and (*B*) [^15^O]H_2_O PET. FFR, fractional flow reserve; MBF, myocardial blood flow.

Heart rate during CMR was higher than during PET in both rest (66 ± 9 vs. 63 ± 10 bpm, *P* < 0.001) and stress (86 ± 12 vs. 84 ± 14 bpm, *P* = 0.031). An increase in heart rate during hyperaemia was comparable between CMR and PET (20 ± 8 vs. 20 ± 11 bpm, *P* = 0.460), presuming an equal response to adenosine. Comparison between MBF measurements of QP-CMR and PET are shown in Table 2. Rest MBF for QP-CMR was higher than for PET (0.98 ± 0.23 vs. 0.93 ± 0.26, *P* < 0.001), whereas stress MBF as measured by QP-CMR was lower than PET (1.64 ± 0.51 vs. 2.73 ± 0.90, *P* < 0.001). Stress MBF was lower in vascular territories with vs. without obstructive CAD for both QP-CMR (1.50 ± 0.50 vs. 1.71 ± 0.50, *P* < 0.001) and PET (2.29 ± 0.76 vs. 2.94 ± 0.89, *P* < 0.001). The correlation coefficient between stress MBF of QP-CMR and PET was 0.34 (*P* < 0.001) with a mean bias of 1.08 ± 0.86 mL·min^−1^·g^−1^ (*Graphical Abstract*). The correlation between FFR and stress MBF was 0.21 (*P* < 0.001) for QP-CMR and 0.45 (*P* < 0.001) for PET (*Figure 2*). The optimal cut-off point for QP-CMR obtained stress MBF to detect hemodynamically significant CAD was found at ≤1.43 mL·min^−1^·g^−1^. Inter-observer variability for QP-CMR stress MBF showed a mean bias of 0.12 (±0.25) mL·min^−1^·g^−1^, with inter-observer reliability for absolute agreement of 0.83 (see Supplementary data online, *Figure S1*).

**Table 2:** MBF measurements of QP-CMR and [^15^O]H_2_O PET

	MBF (mL·min^−1^·g^−1^)		
	QP-CMR	[^15^O]H_2_O PET	*P*-value
Rest MBF (mL·min^−1^·g^−1^)			
All vessels	0.98 ± 0.23	0.93 ± 0.26	< 0.001
Non-obstructed vessels	0.98 ± 0.21	0.92 ± 0.26	0.006
Obstructed vessels	0.99 ± 0.26	0.93 ± 0.27	0.055
*P*-value	0.537	0.666	
Stress MBF (mL·min^−1^·g^−1^)			
All vessels	1.64 ± 0.51	2.73 ± 0.90	< 0.001
Non-obstructed vessels	1.71 ± 0.50	2.94 ± 0.89	< 0.001
Obstructed vessels	1.50 ± 0.50	2.29 ± 0.76	< 0.001
*P*-value	< 0.001	< 0.001	

CMR, cardiac magnetic resonance imaging; MBF, myocardial blood flow; PET, positron emission tomography; QP-CMR, quantitative perfusion CMR.

### Diagnostic performance of QP-CMR, v-CMR and PET

A comparison of the diagnostic performance of QP-CMR, v-CMR, and PET is shown in Table 3. On a per-patient level, QP-CMR showed similar sensitivity (66%) as compared to v-CMR (67%) and lower as compared to PET (80%, *P* = 0.015). Specificity, PPV and NPV did not differ for QP-CMR (60, 74, and 51%, respectively), v-CMR (62, 76, and 52%), and PET (63, 79, and 65%). QP-CMR showed similar overall diagnostic accuracy (64%) and AUC (0.66) as compared to both v-CMR (66% and 0.67, respectively) and PET (74% and 0.78).

**Table 3:** Diagnostic performance per patient and per vessel

	% (95% CI)					
	QP-CMR	v-CMR	*P*-value*	[^15^O]H_2_O PET	*P*-value**	*P*-value***
Per-patient						
Sensitivity	66 (56–76)	67 (57–77)	0.999	80 (71–88)	*0*.*015*	0.036
Specificity	60 (46–74)	62 (48–75)	0.999	63 (49–76)	0.839	0.999
PPV	74 (67–81)	76 (65–84)	0.817	79 (70–87)	0.279	0.409
NPV	51 (42–60)	52 (39–65)	0.806	65 (50–78)	0.025	0.052
Accuracy	64 (56–72)	66 (57–73)	0.902	74 (66–81)	0.044	0.119
AUC	0.66 (0.58–0.74)	0.67 (0.59–0.75)	0.840	0.78 (0.70–0.84)	0.028	0.046
Per vessel						
Sensitivity	54 (45–63)	46 (37–55)	0.215	73 (64–80)	*<0*.*001*	*<0*.*001*
Specificity	68 (63–74)	81 (76–85)	*0*.*003*	66 (60–71)	0.486	*<0*.*001*
PPV	45 (39–50)	53 (44–63)	0.080	50 (43–57)	0.127	0.431
NPV	76 (72–79)	76 (71–81)	0.947	84 (78–88)	*<0*.*001*	*0*.*003*
Accuracy	64 (59–68)	70 (65–74)	0.064	68 (63–73)	0.189	0.506
AUC	0.63 (0.58–0.67)	0.67 (0.62–0.72)	0.246	0.74 (0.70–0.78)	*0*.*001*	0.033

Bonferroni correction applied: statistical significance at *P*-value < 0.0167. Significant *P*-values are marked *in italic.*

AUC, area under the receiver operating characteristic curve; CI, confidence interval; NPV, negative predictive value; PPV, positive predictive value; v-CMR, visually assessed first-pass stress perfusion CMR.

*P-value between QP-CMR and v-CMR. **P-value between QP-CMR and PET. ***P-value between v-CMR and PET.

On a per-vessel level, QP-CMR showed similar sensitivity (54%) and NPV (76%) as compared to v-CMR (46 and 76%, respectively), and lower as compared to PET [73% (*P* < 0.001) and 84% (*P* < 0.001)]. Specificity for QP-CMR (68%) was lower than for v-CMR [81% (*P* = 0.003)], and similar to PET (66%), whereas PPV was similar for all modalities (QP-CMR: 45%, v-CMR: 53%, and PET: 50%). QP-CMR showed similar diagnostic accuracy (64%) as compared to v-CMR (70%) and PET (68%). The AUC of QP-CMR (0.63) did not differ from v-CMR (0.67) and was lower than PET [0.74 (*P* < 0.001)].

### Diagnostic performance stratified by MVD and scar

**Figure 3:**
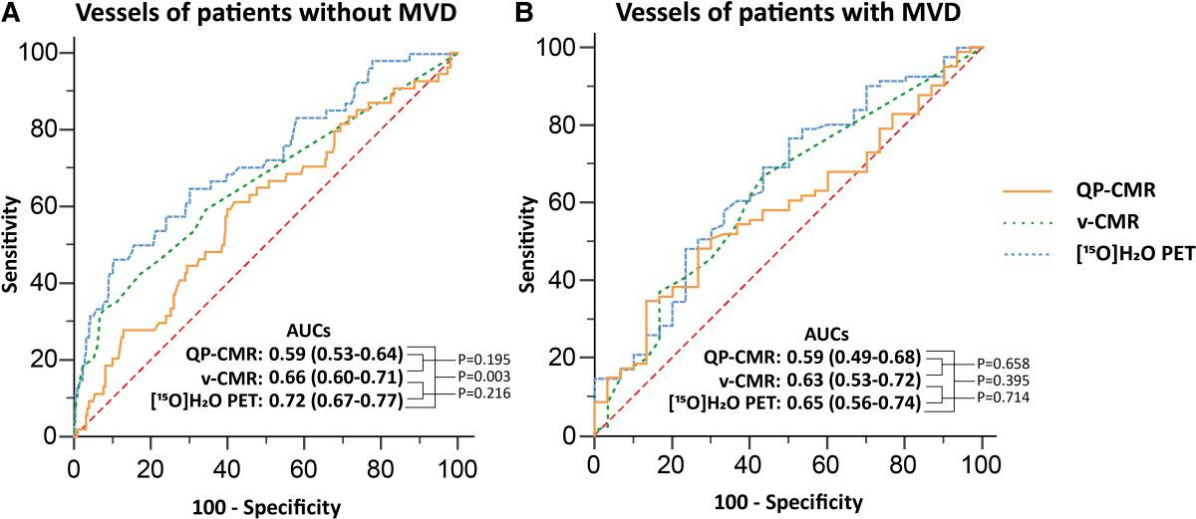
Diagnostic performance in vessels of patients with and without MVD. AUCs of QP-CMR, v-CMR and [^15^O]H_2_O PET in vessels of patients with (*A*) no CAD or 1-vessel disease, and (*B*) two- or three-vessel disease. Bonferroni correction applied: statistically significance at *P*-value < 0.0167. AUC, area under the receiver operating characteristic curve; CAD, coronary artery disease; MVD, multivessel disease.

**Figure 4:**
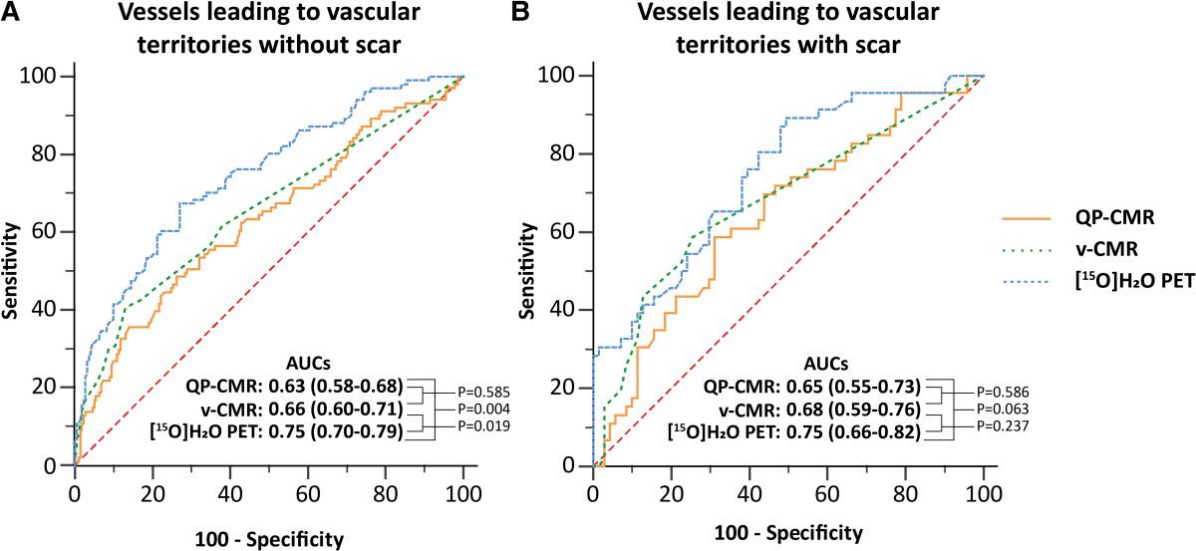
Diagnostic performance in vessels leading to vascular territories with and without scar. AUCs of QP-CMR, v-CMR, and [^15^O]H_2_O PET in vessels leading to vascular territories (*A*) without and (*B*) with scar. Bonferroni correction applied: statistical significance at *P*-value <0.0167.

Per-vessel subanalyses of patients with and without MVD and scar are shown in Tables 4 and 5 and *Figures 3* and *4*. In patients without MVD, diagnostic accuracy of QP-CMR (66%) was lower than v-CMR [77% (*P* = 0.005)] and comparable to PET (67%). Conversely, the AUC of QP-CMR (0.59) was comparable to v-CMR (0.66) and lower than PET [0.72 (*P* = 0.003)]. For patients with MVD, sensitivity and PPV of QP-CMR and PET increased, whereas specificity and NPV decreased. Diagnostic accuracy of QP-CMR (57%) was comparable to v-CMR (51%) and lower than PET [69% (*P* = 0.016)]. For QP-CMR and v-CMR, the overall accuracy decreased, whereas accuracy did not change for PET (Table 4 and *Figure 3*). When stratified by vascular territories with and without CMR-derived scar, overall diagnostic performance was similar for each perfusion modality. Sensitivity and PPV of QP-CMR and PET increased in territories with scar and decreased in territories without scar (Table 5 and *Figure 4*).

**Table 4:** Per-vessel diagnostic performance in patients with and without MVD

	% (95% CI)					
	QP-CMR	v-CMR	*P*-value*	[^15^O]H_2_O PET	*P*-value**	*P*-value***
Vessels of patients without multivessel disease (*n* = 315)						
Sensitivity	44 (31–59)	50 (36–64)	0.547	65 (51–77)	*0*.*011*	0.065
Specificity	71 (65–76)	82 (77–87)	*0*.*013*	68 (62–73)	0.504	*0*.*001*
PPV	24 (18–31)	37 (26–49)	0.017	30 (22–39)	0.105	0.149
NPV	86 (83–89)	89 (84–93)	0.131	90 (85–94)	0.026	0.441
Accuracy	66 (61–72)	77 (72–81)	*0*.*005*	67 (62–72)	0.779	*0*.*008*
Vessels of patients with multivessel disease (*n* = 114)						
Sensitivity	60 (49–71)	43 (33–55)	0.052	78 (67–86)	*0*.*003*	*< 0*.*001*
Specificity	48 (30–67)	71 (52–86)	0.038	47 (28–66)	0.849	0.031
PPV	76 (68–82)	80 (65–90)	0.422	80 (69–88)	0.328	0.961
NPV	31 (22–42)	32 (21–44)	0.903	44 (26–62)	0.079	0.071
Accuracy	57 (47–66)	51 (41–60)	0.330	69 (60–78)	*0*.*016*	*0*.*001*

Bonferroni correction applied: statistically significance at *P*-value <0.0167. Significant *P*-values are marked *in italic.*

*P-value between QP-CMR and v-CMR. **P-value between QP-CMR and PET. ***P-value between v-CMR and PET.

**Table 5:** Per-vessel diagnostic performance in vascular territories with and without CMR-derived scar

	% (95% CI)					
	QP-CMR	v-CMR	*P*-value *	[^15^O]H_2_O PET	*P*-value**	*P*-value***
Vessels leading to vascular territories without scar (*n* = 311)						
Sensitivity	51 (40–61)	47 (37–58)	0.667	64 (53–74)	*0*.*013*	*0*.*014*
Specificity	70 (63–76)	82 (76–87)	*0*.*015*	71 (64–76)	0.861	*0*.*009*
PPV	41 (34–48)	52 (43–61)	0.062	47 (41–54)	0.113	0.380
NPV	77 (73–81)	79 (75–282	0.519	83 (78–86)	*0*.*013*	0.140
Accuracy	64 (58–69)	72 (66–77)	0.047	69 (63–74)	0.180	0.360
Vessels leading to vascular territories with scar (*n* = 118)						
Sensitivity	61 (45–75)	43 (29–59)	0.085	89 (76–96)	*< 0*.*001*	*< 0*.*001*
Specificity	66 (53–76)	79 (68–88)	0.063	51 (39–63)	0.089	*< 0*.*001*
PPV	53 (43–62)	57 (43–70)	0.602	54 (48–60)	0.846	0.684
NPV	72 (64–80)	69 (62–74)	0.462	88 (75–94)	*0*.*005*	*< 0*.*001*
Accuracy	64 (54–72)	65 (56–74)	0.778	66 (56–74)	0.685	0.921

Bonferroni correction applied: statistically significance at *P*-value < 0.0167. Significant *P*-values are marked *in italic.*

*P-value between QP-CMR and v-CMR. **P-value between QP-CMR and PET. ***P-value between v-CMR and PET.

### Prognostic value of QP-CMR-, v-CMR-, and [^15^O]H_2_O PET-defined ischaemia

**Figure 5:**
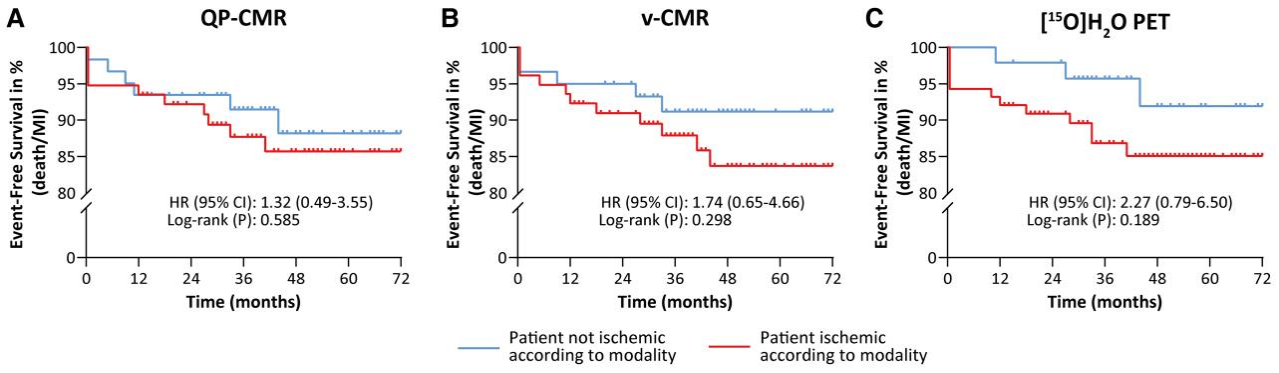
Prognostic value of QP-CMR-, v-CMR-, and [^15^O]H_2_O PET-defined ischaemia. Kaplan–Meier curves of event-free survival of death and MI, based on the presence or absence of ischaemia according to (*A*) QP-CMR, (*B*) v-CMR, and (*C*) [^15^O]H_2_O PET. CI, confidence interval; HR, hazard ratio; MI, myocardial infarction.

Follow-up was achieved in 138 (95%) patients. During a median follow-up period of 44 [32–62] months for QP-CMR and v-CMR, and 44 [32–61] months for PET, 16 (12%) patients experienced an endpoint [all-cause death: *n* = 5 (4%) and non-fatal MI: *n* = 11 (8%)]. Kaplan–Meier survival curves are presented in *Figure 5*. Ischaemia defined by QP-CMR, v-CMR, and PET was not associated with outcome [QP-CMR: HR 1.32 (95% CI: 0.49–3.55; log-rank *P*-value = 0.585), v-CMR: HR 1.74 (CI: 0.65–4.66; *P* = 0.298), and PET: HR 2.27 (CI: 0.79–6.50; *P* = 0.189)].

## Discussion

This substudy of the PACIFIC-2 trial is the first head-to-head comparison between dual-sequence quantitative perfusion CMR, visually assessed first-pass stress perfusion CMR and [^15^O]H_2_O PET perfusion imaging for the detection of FFR-defined hemodynamically significant CAD in patients with a history of MI and/or PCI (*Graphical Abstract*). Our results show a poor agreement between QP-CMR and [^15^O]H_2_O PET-derived stress MBF. Moreover, a quantitative analysis of CMR perfusion imaging does not outperform expert visual assessment, and results in equal diagnostic performance when compared to [^15^O]H_2_O PET.

### Diagnostic performance of QP-CMR

To the best of our knowledge, this is the first study to assess the diagnostic performance of QP-CMR solely in patients with prior CAD. Prior studies showed higher diagnostic performance of QP-CMR.^13,17–19^ Biglands *et al.*^17^ demonstrated an AUC of 0.89 with sensitivity of 87.5% and specificity of 84.5% in 128 patients. Hsu *et al.*^13^ demonstrated similar performance in a smaller retrospective study including 80 patients. Notably, both studies used an anatomical reference standard instead of FFR, impeding accurate comparison to our findings. In addition, both studies defined ischaemia based on the lowest segmental MBF per vascular territory, whereas we averaged MBF of the entire territory to even out delineation errors and to avoid the inclusion of isolated segments with LGE. This strategy was chosen to reduce false positive cases that could result from low segmental MBF due to the possible inclusion of extracardiac structures with low SI or segments with LGE. In addition, the high prevalence of disease in a high-risk population (prior CAD) may provide a disbalance in diagnostic values favouring a sensitive grading at a cost of higher rates of false positive findings in comparison to a ‘prior CAD naive population’. Concordantly, prior studies have shown that the inclusion of patients with prior CAD hampers the diagnostic performance of MPI.^3,11,20^ Although Biglands *et al.*^20^ did not document cardiac history, their study population derived from the CE-MARC trial, with a relatively low occurrence of prior PCI (5%) or MI (8%). Hsu *et al.* had a 21% rate of prior PCI, without mentioning prior MI.

### QP-CMR vs. v-CMR and [^15^O]H_2_O PET

In our study, although the sensitivity of [^15^O]H_2_O PET exceeded that of both CMR modalities, overall diagnostic performance of QP-CMR, v-CMR, and [^15^O]H_2_O PET was comparable. Current literature on the differences between QP-CMR and v-CMR is disparate. In accordance with our results, the majority of available studies have shown a comparable performance of QP-CMR and v-CMR.^17,21–23^ One study found that QP-CMR could outperform v-CMR, mainly due to superior specificity.^24^ However, the overall accuracy of v-CMR in this study was lower compared to other studies with a comparative study population, and visual assessment was not performed by a dedicated core lab.^25^ All in all, our results indicate that the use of QP-CMR does not seem to have an additional value over visual assessment of stress perfusion CMR in a population with prior CAD. Furthermore, we compared QP-CMR with [^15^O]H_2_O PET, using FFR as a reference standard. To the best of our knowledge, our study is the first to comprehensively compare QP-CMR and [^15^O]H_2_O PET in this manner, utilizing a dual-sequence protocol and automated post-processing software to streamline clinical applicability. The correlation of stress MBF between both modalities observed in our study is lower than those reported in the literature.^26–28^ In our patient population with prior CAD, however, a relatively small number of patients would have high perfusion values, leading to a narrow spread in perfusion and subsequently lower correlation. This narrow spread is even more pronounced in the QP-CMR MBF parameters compared to the PET MBF parameters, leading to a large difference in QP-CMR and PET stress MBF. One cause of this difference is the variation in tracer kinetics between GBCA and [^15^O]H_2_O. [^15^O]H_2_O is extracted from the myocardium proportionally linear to perfusion in the myocardium, whereas for GBCA the ratio between myocardial uptake and actual perfusion is not linear and requires extensive correction, increasing the susceptibility to differences in MBF.^29^ Moreover, the studied patient population has a prior MI rate of 52%, and as such, these patients have a relatively large amount of LGE in their myocardium. GBCA accumulates in scarred regions at a lower rate than in healthy myocardium during first-pass perfusion, and these scarred regions are therefore detected as impaired perfusion by the technique of QP-CMR. In contrast, [^15^O]H_2_O typically does not perfuse scarred myocardium and thus does not reflect impaired perfusion in these regions. We have to acknowledge that the cvi42 software does not allow for corrections of key time points on the SI and AIF curves when there is suspicion of an error. Possibly, including an option for such corrections in the automated software could improve concordance with [^15^O]H_2_O PET, though at a cost of analysis time efficiency. Nevertheless, despite the lack of agreement between both modalities, the diagnostic performance of QP-CMR was equal to [^15^O]H_2_O PET, although sensitivity was higher for [^15^O]H_2_O PET. However, it should be noted that in this relatively small sample size, the diagnostic performance of [^15^O]H2O PET was numerically higher on a per-patient level, which could reach significance when tested on a larger sample size. Of note, in 9% of the patients that underwent stress, CMR quantitative analysis could not be performed due to software-related technical difficulties of unknown origin which warrant further investigation. The observed differences in diagnostic performance could partially be explained by the difference in LV spatial covering between QP-CMR and [^15^O]H_2_O PET, and the relatively small difference in QP-CMR stress MBF between obstructed and non-obstructed vessels in comparison to the difference in PET stress MBF. Moreover, FFR has originally been validated against [^15^O]H_2_O PET, potentially biasing outcomes in favour of [^15^O]H_2_O PET over QP-CMR.^30^ As well, it is important to acknowledge that our findings may not directly apply to all PET tracer modalities, as tracer kinetics vary between PET modalities. Although [^15^O]H_2_O is generally acknowledged as the gold standard for myocardial flow assessment, the production of [^15^O]H_2_O requires a cyclotron in close vicinity to the PET scanner, limiting widespread clinical availability.^29^ Finally, we investigated the prognostic value of ischaemia defined by each imaging modality. Ischaemia in the three modalities was not associated with all-cause death or non-fatal MI. However, the results of the present study should be interpreted with caution given the low event rate and overwhelming data regarding the association of ischaemia with MPI and detrimental patient outcome.^31–33^

### QP-CMR in MVD and vascular territories with scar

For PET, quantification of MBF has been shown to have incremental value over visual assessment in the detection of MVD.^34^ Balanced ischaemia accompanying MVD prohibits the detection of a healthy myocardium that can function as a reference for visual assessment. Quantification of MBF provides values independent of surrounding areas, rendering a healthy reference superfluous. However, during CMR assessment of a patient with MVD, the epicardium can be used as a healthy reference, and balanced ischaemia only hinders visual assessment in the case of three-vessel transmural ischaemia. Indeed, our study demonstrated comparable diagnostic accuracy between QP-CMR and v-CMR in patients with MVD. These results are in line with the previously mentioned CE-MARC substudy.^17^ Contrarily, Kotecha *et al.*^9^ report an incremental value of QP-CMR for correctly identifying obstructed vessels of MVD patients in a population with a relatively high amount of two-vessel (*n* = 48) and three-vessel (*n* = 47) disease. Possibly, the sample size in our study was not sufficient to demonstrate significant differences between QP-CMR and v-CMR in MVD. Furthermore, we assessed the diagnostic performance of MPI in vascular territories with CMR-derived scar. These territories are deemed highly challenging for non-invasive imaging evaluation due to the mixed presence of scarred and viable tissue, with subsequent influence on the determination of which areas should be classified as ischaemic. Moreover, slowed wash-in kinetics of GBCA in infarcted areas reduces QP-CMR-derived MBF leading to a false diagnosis of ischaemia. Hence, vascular territories with scar are often excluded from analysis.^8,28^ In the present study, we averaged the MBF of entire vessels to ensure that ischaemia was not defined solely by MBF values from scarred segments. Using this method, analysis of vessels with scar did not alter the overall diagnostic performance of QP-CMR, indicating that QP-CMR can be used in a clinical population without the necessity of excluding patients based on scar presence.

### Limitations

The present data should be considered in light of the following limitations. First, our study sample may limit statistical power for diagnostic performance assessment and is not powered for clinical endpoints. Second, we used a dual-sequence scanning protocol in which GBCA dose was higher than in other dual-sequence QP-CMR studies to reflect clinical practice, where a higher dose is used to facilitate visual CMR assessment.^7,9,13,18^ Higher contrast doses could lead to underestimation of AIF,^12^ thus influencing diagnostic accuracy. Different GBCA administration schemes, advanced scanning sequences, or a dual-bolus technique may yield higher diagnostic accuracy of QP-CMR in this patient population. Third, dedicated core laboratories performed v-CMR and PET image analysis, whereas QP-CMR analysis was not performed by a core laboratory. Although QP-CMR is an automated process, delineation errors have to be manually adjusted. We aimed to minimize the impact of possible incorrect manual adjustments by averaging MBFs of an entire vascular territory for diagnostic accuracy calculations. This approach also helped reduce the risk of defining ischaemia solely based on scarred segments. However, averaging MBF might increase the risk of false negatives, and different analysis methods could yield varying results. Fourth, this study lacks other quantitative perfusion parameters—such as the myocardial perfusion reserve, the relative flow reserve or transmural MBF ratios—and invasive microvascular assessment. Fifth, differences between the AHA 17-segment model and true anatomy may have occurred and could have influenced per-vessel subanalyses. Sixth, 83% of our study population was male. The results of this study might therefore not be directly extrapolated to females. Seventh, non-invasive imaging modalities were compared to FFR-defined CAD. Although FFR is the reference standard for revascularization decision-making, the impact of coronary microvascular dysfunction and scar on these physiological measurements may vary. Finally, vessels with a subtotal lesion or chronic total occlusion were deemed to have significant CAD and were assigned an FFR value of 0.50; however, the true hemodynamically significance has not been invasively confirmed.

## Conclusion

In this head-to-head comparative study in patients with a history of MI and/or PCI, dual-sequence quantitative perfusion CMR and [^15^O]H_2_O PET-derived stress MBF demonstrated poor agreement. Despite this poor agreement, the diagnostic performance of quantitative perfusion CMR was equal to that of quantitative [^15^O]H_2_O PET for the detection of hemodynamically significant CAD as defined by FFR. Moreover, quantitative perfusion CMR and expert v-CMR yielded equal diagnostic performance as well, questioning the additive value of CMR stress perfusion quantification in this population.

## Supplementary data

Supplementary data are available at *European Heart Journal - Cardiovascular Imaging* online.

## Supplementary Material

jeae262_Supplementary_Data

## Data Availability

The data underlying this article will be shared on reasonable request to the corresponding author.
