# Supplementary material for: Diagnostic performance of quantitative perfusion cardiac magnetic resonance imaging in patients with prior coronary artery disease
Source: Eur Heart J Cardiovasc Imaging. 2024 Oct 9;26(2):207–17. doi: 10.1093/ehjci/jeae262 (PMC11781829; doi:10.1093/ehjci/jeae262)
Supplement: jeae262_Supplementary_Data [file jeae262_supplementary_data.docx]

**Supplemental information**

Diagnostic Performance of Quantitative Perfusion Cardiac Magnetic Resonance Imaging in Patients with Prior Coronary Artery Disease

**Figures**

## Supplemental Figure 1: Bland-Altman analysis of inter-observer variability of QP-CMR


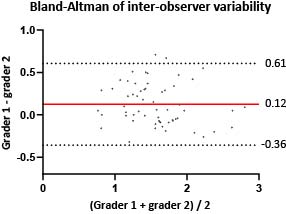


**Supplemental Figure 1. Bland-Altman analysis of inter-observer variability of QP-CMR.** Bland-Altman plot demonstrating inter-observer level of agreement of QP-CMR stress MBF with mean bias ± 1.96 SD. Abbreviations: QP-CMR: quantitative perfusion cardiac magnetic resonance imaging, MBF: myocardial blood flow, SD: standard deviation.
